# Supplementary material for: Evolution of Attenuation and Risk of Reversal in Peste des Petits Ruminants Vaccine Strain Nigeria 75/1
Source: Viruses. 2019 Aug 7;11(8):724. doi: 10.3390/v11080724 (PMC6724400; doi:10.3390/v11080724)
Supplement: Supplementary file 1 [file viruses-11-00724-s001.pdf]

Supplementary material

Table S1: Primer set used for long fragment analysis of the PPRV genome

**Fragment 1 (4050 bp)**
PPRV-3F
CAAACAAAGTTGGGTAAGGA
PPRV-4053R
TTCTCCCATGAGCCGACTATGTA
**Fragment 2 (3663 bp)**
PPRV-3394F
TATTACAAAAAACTTAGGAGCAAGGG
PPRV-7057R
CAGCAGCATATTAATGTGACAAG
**Fragment 3 (3816 bp)**
PPR-6025F
CTTGCAAATAAGGAGACCATACT
PPRV-9841R
GGCATATATGGGACTGTTCTTT
**Fragment 4 (3800 bp)**
PPRV-9247F
CACAACCCAGCCAACAATGTTAT
PPRV-13066R
GCTAGCATCCAAGCTTCACTCCATGA
**Fragment F5 (3335 bp)**
PPR-12607F
CGCCTTTCCAATTATGATTA
PPRV-15942R
GAGACAAAGCTGGGAATAGA

Table S2: Pairwise Manhattan distances calculated based on the base frequencies and the consensus sequences (diagonal from top/left to bottom/right highlighted in bold), the base frequencies in the populations (above the diagonal), or the consensus sequences (below the diagonal), respectively

	WTcons	P47CHS1	P50	P58	P62	P65	P67	P69	P70	P75	Vaccin	P75C5	P75C10	P80	P85	P90	P99	P130	P149
**WTcons**	**0**	49.7	39.8	62.7	52.9	53.8	46.1	61.2	72.7	58.7	59.2	71.1	59.4	50.8	60.9	60.5	61.4	69.1	62.9
**P47CHS1**	28	**32.6**	36.4	43.8	41.9	39.3	35.3	48.4	53.5	34.9	40.9	54.4	43.9	49.8	38.1	52.2	48.6	48.9	48.8
**P50**	24	4	**16.1**	43.5	36.6	31.3	24.2	46.1	54.01	40.3	39.9	53.9	42.2	34.4	43.6	41.5	46.9	53.7	45.3
**P58**	32	12	8	**36.7**	34.4	31.4	32.6	29.3	44.7	25.7	26.2	47.3	37.6	48.1	38.3	54.5	38.9	49.3	44.2
**P62**	32	12	8	4	**25.5**	26.6	22.2	32.9	42.5	27.1	27.5	41.6	30.04	34.6	31.6	45.9	33.29	41.8	34.4
**P65**	32	12	8	4	0	**22.6**	17.2	32.3	44.7	29.9	30.3	43.7	32.1	33.3	32.7	43.1	36.1	42.2	34.8
**P67**	32	12	8	4	0	0	**14.3**	31.23	38.9	25.03	24.8	39.4	27.4	24.3	28.8	34.7	31.7	38.5	30.4
**P69**	32	12	8	4	0	0	0	**37.3**	35.1	25.1	26.7	47.7	36.7	44.8	39.1	51.6	34.4	49.7	43.2
**P70**	32	12	8	4	0	0	0	0	**44.9**	30.7	39.1	59.3	49.4	52.5	43.7	51.8	48.2	51.9	53.8
**P75**	32	12	8	4	0	0	0	0	0	**30.9**	17.2	38.6	28.5	40.3	23.4	38.7	29.8	34.9	35.9
**Vaccin**	32	12	8	4	0	0	0	0	0	0	**31.7**	36.8	23.7	33.9	28.4	47.3	31.003	42.5	36.3
**P75C5**	46	26	22	18	14	14	14	14	14	14	14	**38.2**	24.1	47.5	29.1	61.3	46.9	54.3	48.6
**P75C10**	42	22	18	14	10	10	10	10	10	10	10	4	**28.3**	30.7	22.8	51.1	36.1	43.7	37.01
**P80**	40	20	16	12	8	8	8	8	8	8	8	10	6	**23.6**	36.3	41.02	46.1	1	44.9
**P85**	38	18	14	10	6	6	6	6	6	6	6	8	4	2	**31.2**	44.3	38.8	36.3	37.5
**P90**	34	14	10	6	2	2	2	2	2	2	2	16	12	6	8	**34.7**	48.01	47.9	51.8
**P99**	38	18	14	10	6	6	6	6	6	6	6	20	16	14	12	8	**36.9**	41.1	32.4
**P130**	48	28	24	20	16	16	16	16	16	16	16	30	26	24	22	18	10	**40.45**	28.4
**P149**	44	24	20	16	12	12	12	12	12	12	12	26	22	20	18	14	6	4	**23.1**

P47CHS1: Passage 47 on Vero cells + one passage on CHS cells; P50: 50th passage on Vero cells (like other Ps… in these plots)

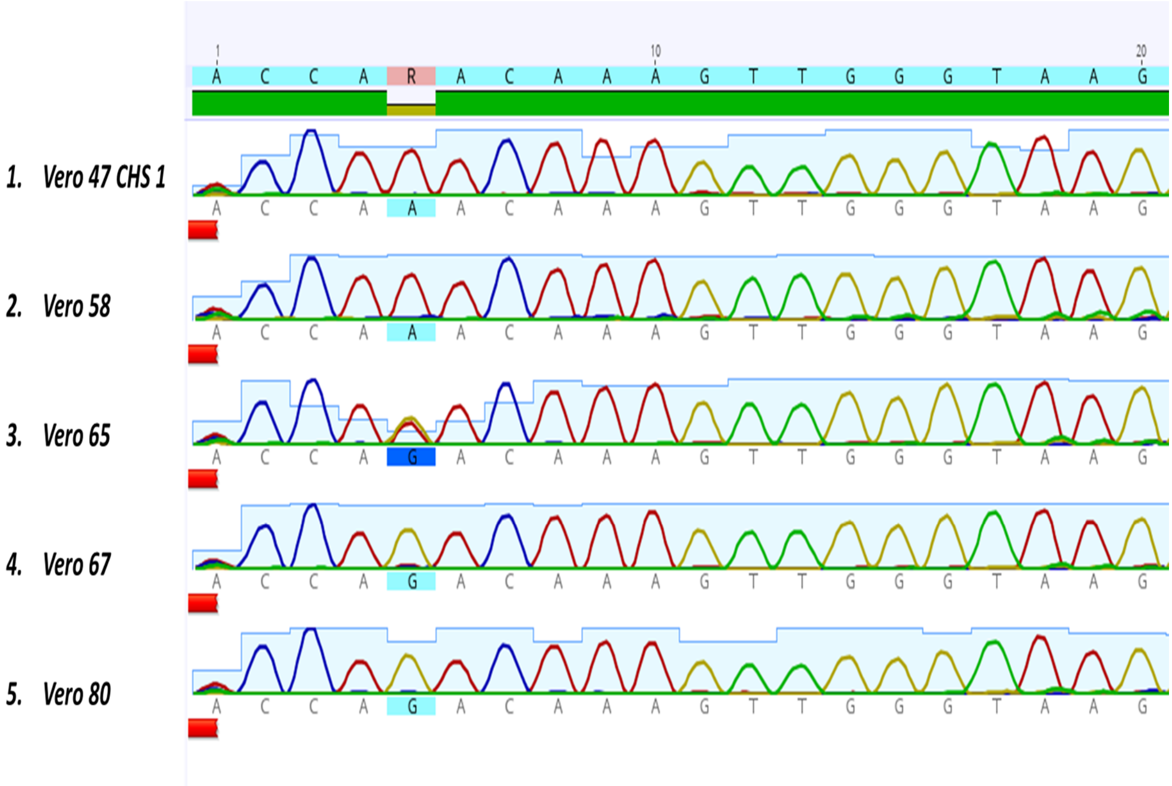

Figure S1: Chromatogram alignment of the RACE PCR Sequencing. As can be seen in the figure, we observed a double peak on the 5th position of the 65th passage on Vero cells.

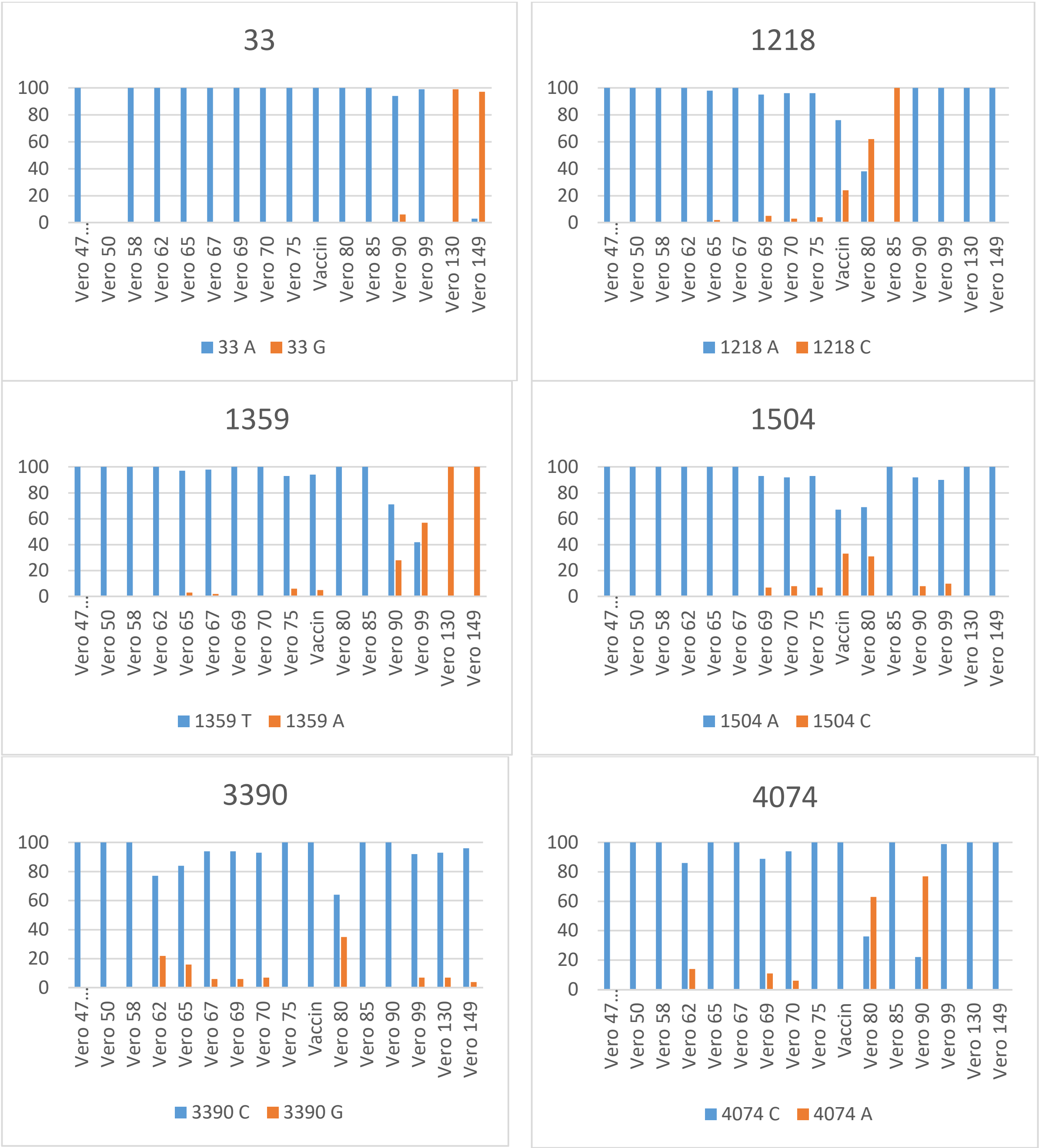

Figure S2: Variants appeared and/or disappeared during cell passages. The number at the top of each histogram shows its position in the genome. Orange bars represent the alternative variant, blue bars represent the wild version.
